# A Diagnostic Challenge of Oral Psoriasis in a Pediatric Patient: A Case Report and Review of the Literature

**DOI:** 10.7759/cureus.49572

**Published:** 2023-11-28

**Authors:** Arnesh Garg, Aravind Warrier S, K Subadra

**Affiliations:** 1 Sri Ramachandra Dental College and Hospital, Sri Ramachandra Institute of Higher Education and Research, Chennai, IND; 2 Oral Medicine and Radiology, Sri Ramachandra Institute of Higher Education and Research, Chennai, IND

**Keywords:** pediatric psoriasis, whole exome sequencing, autosomal recessive, genetic disorders, psoriasis

## Abstract

Congenital psoriasis is a rare skin disease that can clinically manifest in the oral cavity in many ways. Although manifestations over the skin are frequent, oral manifestations are rare, especially in pediatric patients. A clear family history, proper examination, and investigations are essential to diagnose this condition. This case report aims to highlight the oral and systemic manifestations of a case of psoriasis in a male pediatric patient.

## Introduction

Psoriasis is a chronic, remitting, and relapsing autoimmune inflammatory disorder that most commonly affects the scalp, elbows, knees, intergluteal region, and abdomen [1]. The mean age of onset for the first presentation of psoriasis can range from 15 to 20 years [2]. Psoriasis in the oral cavity can be classified into a spectrum of four variants based on their clinical features [3]. A diagnosis is made by correlating the history and clinical manifestations and confirmed by histopathological analysis. Genetic testing and pedigree charts play a significant role in detecting and ruling out various genetic diseases. Treatment of oral psoriasis involves topical and systemic therapies. In this case report, we present the case of a young male who presented with a complaint of a white patch in the oral cavity, highlighting its clinical features, differential diagnosis, and oral and systemic implications.

## Case presentation

**Figure 1:**
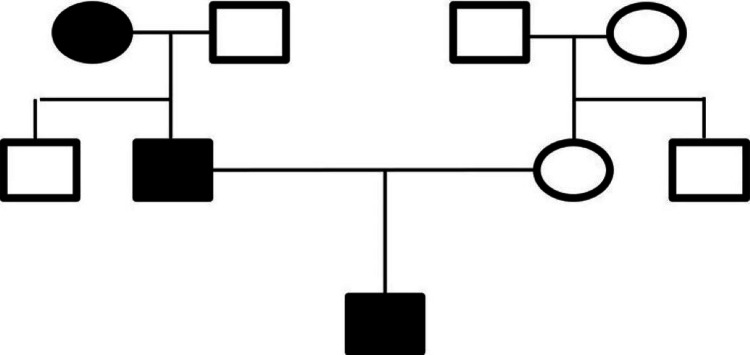
A pedigree chart describing the affected individuals in different generations.

A 13-year-old male presented with a complaint of a white patch on his cheeks and tongue for the past 12 years. The patient gave a history of the lesion being present for the past 12 years which had increased to the present size with a constant burning sensation. On further probing his medical history, a skin biopsy was taken two years ago with features of spongiotic dermatitis. Hematological analysis showed increased IgE levels of over 30,000 IU/mL. Ultrasound of the abdomen revealed an ectopic left kidney. Further, he provided a history of first-degree consanguineous marriage. A pedigree chart was created from the history which showed that previous generations also suffered from similar symptoms over their skin and oral cavity (Figure 1).

**Figure 2:**
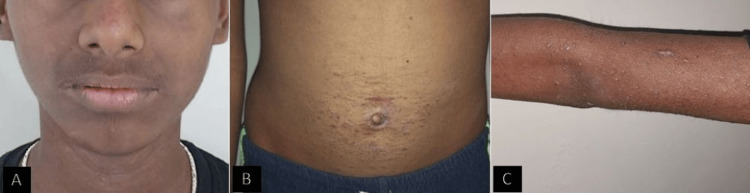
Multiple papules seen over the face, arms, and torso.

**Figure 3:**
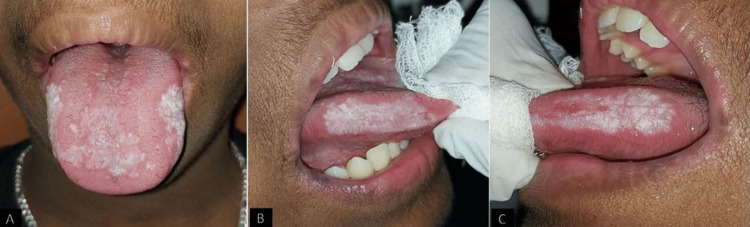
Keratotic patches over the dorsum and lateral borders of the tongue.

**Figure 4:**
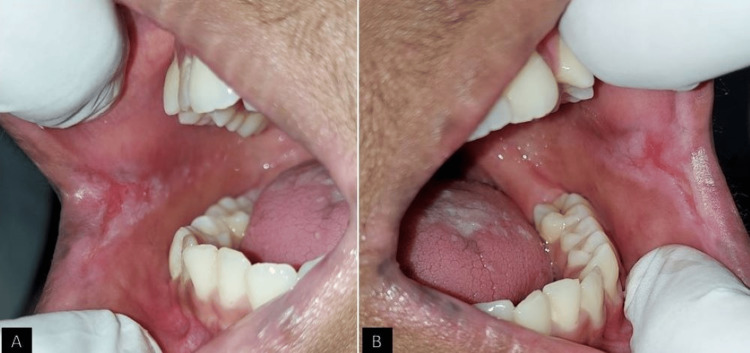
Keratotic patch with erythematous component on the right and left buccal mucosa.

On examination, multiple extraoral scaly papules were seen over his arms, torso, and face with continuous itching over the affected area (Figure 2). Intraorally, a thick, hyperkeratotic plaque was evident bilaterally over the lateral borders and the dorsum of the tongue (Figure 3). Similar lesions were also seen bilaterally in the buccal mucosa accompanied by an erythematous component (Figure 4). The patient gave a history of a burning sensation with a visual analog scale of 3. On palpation, the lesions were non-scrapable and non-tender.

Based on the medical history, family history, and correlating it with the intraoral findings, a provisional diagnosis of atrophic candidiasis was made for the oral lesion, whereas a differential diagnosis of dyskeratosis congenita and Job’s syndrome with idiopathic leukoplakia was made by correlating the systemic manifestations.

**Figure 5:**
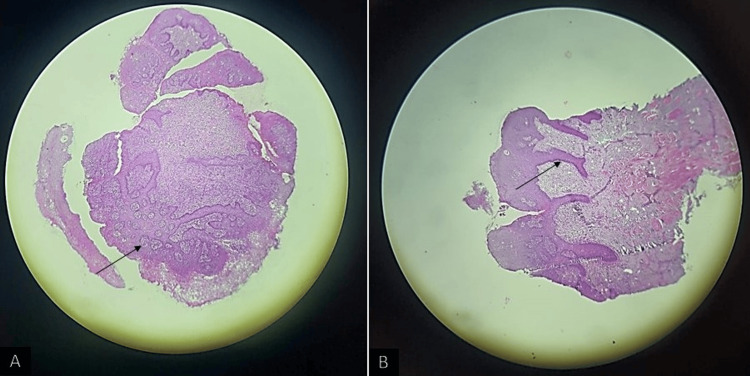
10× view revealing keratotic squamous epithelium with microabscesses and elongated rete ridges.

Further, an incisional tongue biopsy on the lateral border aspect was performed. Histopathological features under 10× view revealed keratotic squamous epithelium with elongated rete ridges and plenty of intraepithelial microabscesses with neutrophils suggestive of psoriasis (Figure 5). Whole exome sequencing showed a homozygous missense variation in the *IL17RA* gene in exon 13 of homozygous zygosity suggestive of autosomal recessive traits (Table 1). By correlating these investigations with the clinical findings, a final diagnosis of oral psoriasis was made.

**Table 1:** Whole genome sequencing revealing a heterogeneous compound suggestive of an autosomal recessive disorder.

Gene variant details	Zygosity	In silico tools	MAF	OMOM disease	Inheritance
CIITA(+) c.2804T>C	Likely compound heterozygous	PolyPhen-2-PrD; SIFT-D	1000G-NA;gnomAD:NA;MedVar-0.002%	Bare lymphocyte syndrome type 2	Autosomal recessive

The patient was prescribed antioxidant supplements for one month and topical clotrimazole for two weeks. In addition, he was also advised an antioxidant-rich diet. The patient was also referred to the Department of Dermatology for further management of the rash over the skin. On evaluation of the patient after a month, the itching had reduced and the extent of the white patches in the oral cavity had mildly reduced. The patient was asked to continue the medication and is currently under follow-up.

## Discussion

Genetic and dermatological diseases can manifest in the oral cavity in many ways that resemble each other. This makes it challenging to diagnose based on the clinical findings alone. Psoriasis can have features that can depict both genetic diseases and dermatological disorders.

Psoriasis is a chronic, remitting, and relapsing autoimmune inflammatory disorder that affects the skin and oral cavity with a strong genetic predisposition. The word “psoriasis” was first coined in 1808 by Robert Wilan, derived from the word “psoara” of Greek origin, which means “to itch.” It most commonly affects the scalp, elbows, knees, intergluteal region, and abdomen, similar to our case presentation [1].

Approximately 1-3% of the global population is affected by psoriasis in their early lives [1]. The mean age of onset for the first presentation of psoriasis can range from 15 to 20 years of age, with a second peak occurring at 55-60 years. Psoriasis is classified into two types based on familial history. Type 1 psoriasis has a positive familial history, starts at an early age of before 40 years, and is associated with the gene *HLA-Cw6*. Type 2 psoriasis does not show a familial history, presents after 40 years of age, and is not associated with *HLA-Cw6* [2]. A study showed that the onset of pediatric psoriasis in boys was between 6 and 10 years of age, while girls showed symptom onset between the ages of 10 and 14 years [4]. Congenital psoriasis may be passed on by first- or second-degree consanguineous marriages and can be tracked by constructing a pedigree chart showing the lineage and other members with similar conditions. In our case, the lesions were seen in individuals across three different generations, with symptoms arising from less than three years of age, suggesting that the disease might be hereditary in nature.

In 1986, Van der Wall and Pindborg classified psoriasis in the oral cavity into the following four clinical variations: well-defined small roundish lesions that are gray to yellowish-white; lacy, elevated white lesions of the oral mucosa, paralleling cutaneous lesions; fiery-red erythematous oral lesions that are associated with acute cutaneous lesions; and benign migratory glossitis [3]. Pediatric psoriasis is a relatively common condition [5,6] and is often found in populations of the Middle East, with psoriasis present at birth appearing to be quite rare [7,8]. Given the various dermatological appearances of pediatric psoriasis, several other conditions should be considered in the differential diagnosis such as scaly eruptions, atopic dermatitis, seborrheic dermatitis, dermatophyte or fungal infections such as candidiasis and contact dermatitis can be considered as differential diagnosis [9].

Oral manifestations of psoriasis may resemble other lesions such as oral candidiasis or leukoplakia, in which case a biopsy may be performed to rule out any other lesion [10]. Our patient showed the presence of white patches over the tongue and buccal mucosa which are common sites of leukoplakia. Dyskeratosis congenita is a rare genetic mutation that is characterized by the presence of small scaly papules over the limbs and torso along with leukoplakia in the oral cavity [10]. Our patient presented with a first-degree family history of similar lesions and the clinical features seen in this genetic disorder. Hence, this condition was considered a differential diagnosis. Another reason for considering leukoplakia at such an early age was due to this condition being a differential diagnosis. Job’s syndrome, also known as hyper IgE syndrome, is a condition wherein patients have an abnormally high IgE count, which manifests from an early age [11]. Due to an abnormally high count in our case, this condition was also considered a differential diagnosis.

Genetic testing plays an important diagnostic role in identifying genetic disorders to rule out any underlying genetic condition. Whole exome sequencing is a method used to analyze and determine the variations of all coding regions, or exons, of known genes. The advantage of this method is that it provides coverage of more than 95% of the exons, which contain 85% of disease-causing mutations in Mendelian disorders and many disease-predisposing single-nucleotide polymorphisms throughout the genome [12]. Interleukin 17 is a cytokine that links T-cell activation to neutrophil mobilization and activation and mediates protective innate immunity to pathogens or contributes to the pathogenesis of psoriasis [13]. In our case, there was a pathogenic variant in the IL-17 on the 13th exon on whole exome sequencing, which can be associated with psoriatic lesions.

Treatment of psoriatic lesions can comprise both topical and systemic protocols. The primary line of treatment comprises topical corticosteroids such as triamcinolone and hydrocortisone and immunosuppressants such as cyclophosphamide. The side effects of corticosteroids include a decrease in immunity and opportunistic infections. However, as the side effects of topical steroids are relatively less compared to systemic disorders, the patient did not have any side effects. Certain drugs such as benzydamine may be used in the form of mouthwashes to relieve burning or pain in the case of multiple ulcers. Retinol and retinoic acid, derived from vitamin A, have been shown to promote epithelium formation, thus reducing the size of lesions. Low-level laser ablation can be used to treat the lesions on the epithelial surface [4,14,15]. Other newer lines of treatments with anti-IL-17 biologics have shown promising results in clinical trials for psoriasis and other such conditions [10].

## Conclusions

Oral psoriasis is a rare autoimmune disorder that can manifest in multiple forms and can be challenging to diagnose. These patients may also present with various systemic disorders. Hence, a thorough history, clinical findings, and adequate investigations are needed for a definitive diagnosis after ruling out possibilities. Early diagnosis and timely intervention can help to keep the condition under control.
